# Inequality, validity of self-reported height, and its implications for BMI estimates: An analysis of randomly selected primary sampling units' data

**DOI:** 10.1016/j.pmedr.2019.100974

**Published:** 2019-08-16

**Authors:** Alexi Gugushvili, Ewa Jarosz

**Affiliations:** a Department of Social Policy and Intervention, Nuffield College, University of Oxford, Barnett House, 32 Wellington Square, Oxford OX1 2ER, United Kingdom; b Department of Sociology, Centre for Time Use Research, University of Oxford, Institute of Philosophy and Sociology, Polish Academy of Sciences, 74 Woodstock Road Oxford, OX2 6HP, United Kingdom

**Keywords:** Height, BMI, Income inequality, Multilevel analysis, Life in transition survey

## Abstract

Any systematic errors in self-reported height, a measure commonly used in health research, may produce biased BMI estimates and reduce the effectiveness of public health interventions. To our knowledge, none of the studies evaluating the validity of self-reported height explore this issue in cross-national settings. This study analyses data on a sub-set of 750 individuals with information on self-reported and measured height from the Life in Transition Survey (LITS) conducted in 34 European and Central Asian countries in 2016. We make use of the unique design of LITS in which all respondents reported their height, but in one randomly selected primary sampling unit in each country the actual height was also measured, using a portable stadiometer. In addition to analysing individual-level characteristics, using a multiply imputed dataset for missing data and multilevel mixed-effects regressions, we test if macro-level factors are associated with respondents under- or over-reporting their height. We find that on the aggregate level self-reported and measured height estimates are not statistically different, but some socio-demographic groups such as women and those who live in rural areas are likely to overestimate their height. Adjusting for this bias would lead to the higher estimates of the proportion of individuals who are overweight and obese. The results from multilevel analysis also show that macro-level factors do not per se explain the likelihood of misreporting height, but rather some of the effects of individual characteristics are moderated by income inequality.

## Highlights

• Systematic errors in self-reported height may produce biased BMI estimates.
• On the aggregate level self-reported and measured height are indistinguishable.
• Women and those who live in rural areas are likely to overestimate their height.
• This bias can increase the estimated population BMI level by 2.1%.
• Some of the effects of individual variables are moderated by income inequality.

## 1 Introduction

Quality of self-reported data on individuals' height has implications for social epidemiology and public health. Height, along with weight, is used to calculate individuals' body mass index (BMI) which is one of the major predictors of individuals' health (Calle et al., 1999; Tam and Yeung, 2018). Since a large share of data on height in demographic, social, and health surveys are based on individuals' declarations (Guilcher et al., 2017; Utter et al., 2018), any systematic errors in this measure may produce biased BMI estimates and reduce the effectiveness of public health campaigns that aim to raise awareness of obesity risks. For instance, the analysis of data on self-reported anthropometric measures from participants in the Oxford cohort of the European Prospective Investigation into Cancer and Nutrition showed that 22.4% of men and 18.0% of women were assigned incorrect BMI categories based on self-reported measures (Spencer et al., 2002).

Although self-reported height in most cases is believed to be a quite accurate indicator of actual height (Elgar et al., 2005; Nakamura et al., 1999; Stewart, 1982), some studies find that individuals are likely to over-report their height by as much as 6.9 cm (Brener et al., 2003; Spencer et al., 2002). Existing research in different contexts and countries identified individual-level covariates of the validity of self-reported height such as gender, age, and educational attainment (Ezzati et al., 2006; Hauck et al., 1995; Nieto-García et al., 1990). There is also an indication that those individuals who are socially advantaged are more likely to misreport their height (Danubio et al., 2008), but the exact reasons of this misreporting are unknown. It is speculated that since height is often linked to higher status, those in the higher ranks of social hierarchy, particularly men, want to be associated with this desired physical feature (Toma et al., 2008).

In addition to the above-cited research, there are a number of other studies that evaluate the validity of self-reported height and its individual-level correlates (Elgar et al., 2005; Gorber et al., 2007; Nakamura et al., 1999; Niedhammer et al., 2000; Shin et al., 2014; Zhou et al., 2010), but to our knowledge none of them explore agreement between self-reported and measured height in large cross-national settings. Moreover, previous research does not investigate how the contextual environment in which individuals reside is associated with validity of self-reported height or whether macro-level factors moderate the effects of individual characteristics on height self-reporting. One hypothesis could be that the general levels of economic inequality are associated with the prevalence of bias in declared height. Countries with higher level of economic inequality are shown to be less cohesive and less socially integrated with lower levels of interpersonal trust and higher level of violence (Elgar et al., 2009; Wilkinson and Pickett, 2009a, Wilkinson and Pickett, 2009b), which could be also associated with deceptions in routine survey responses. Inequality can also moderate the influence of socio-economic position on reported height because income inequality has been shown to intensify social comparison among individuals (Cheung and Lucas, 2016).

This study has three main aims: first, to evaluate the accuracy of self-reported height data in cross-national settings; second, to identify if there are systemic differences in misreporting height conditioned by various socio-demographic and socio-economic variables; and third, to test if the macro-level environment – more specifically income inequality – of the countries where individuals reside is associated with under- or over-reporting of height and if macro-level factors moderate the effect of individual characteristics on biased height self-reporting. To address these questions we use data from randomly selected groups of individuals across a large number of societies and compare the values reported in the survey with the values measured with a precision instrument. In the next section, among other findings, we describe the dataset used in this study, the main outcome variable, its individual and macro-level covariates, and the employed statistical methods. In the Results section we show that macro-level factors do not per se explain the likelihood of misreporting height, but rather some of the effects of individual characteristics are moderated by income inequality.

## 2 Method

### 2.1 Data

The present study analyses data from the Life in Transition Survey (LITS) commissioned by the European Bank for Reconstruction and Development (EBRD, 2016) and conducted in 2016 in the following in 34 countries: Albania, Armenia, Azerbaijan, Belarus, Bosnia and Herzegovina, Bulgaria, Croatia, Cyprus, Czechia, Estonia, North Macedonia, Georgia, Germany, Greece, Hungary, Italy, Kazakhstan, Kosovo, Kyrgyz Republic, Latvia, Lithuania, Moldova, Mongolia, Montenegro, Poland, Romania, Russia, Serbia, Slovak Republic, Slovenia, Tajikistan, Turkey, Ukraine, and Uzbekistan. LITS is widely used in comparative social research (Gugushvili, 2019, Gugushvili, 2016; Urbaeva, 2019). Respondents in LITS were selected randomly, using a two-stage sampling procedure. First, the Primary Sampling Units (PSU) were used which are usually electoral districts, polling station territories, census enumeration districts, or other administrative areas. Next, secondary Sampling Units, households, were used. Each country had a minimum of 50 PSUs with each PSU containing at least 20 households.

### 2.2 Reported and measured height

We make use of the unique design of LITS in which all respondents were asked to report their height, but in one randomly selected PSU in each country the actual height was also measured. In the selected PSU the height of respondents was measured without prior warning using a portable stadiometer, which took place after the data on self-reported height was collected. Both self-reported and actual height were given in centimetres and millimetres. Since the outcome variable of this study in multivariate analysis is based on difference between reported and measured height, we use the sub-sample of LITS consisting of 750 individuals. The relatively small number of observations for individual countries does not allow us to generalise findings as nationally representative in these specific countries, but since PSUs where height was measured were randomly selected, we believe that identified patterns are reflective of the reliability of data on self-reported height.

### 2.3 Individual-level covariates

In multivariate analysis of the validity of self-reported height, we use the following socio-demographic and socio-economic variables as potential covariates of misreporting height: respondents' gender is included when analysis is conducted using the pooled sample of men (47.0%) and women (53%); individuals' age (min-max 18:94, mean 48.5, SD 17.3); the type of settlement (urban 62.7%, rural 37.3%); respondents' education (tertiary educational attainment 15.4%, other levels of education 84.6%); marital status (married 61.8%, other marital statuses 38.2%); labour market position (working 47.3%, unemployed 34.7%, out of labour market 18.1%); subjective position on socio-economic ladder (lowest-highest 1:10, mean 4.6, SD 1.8); and two anthropometric measures – self-reported height (min-max 140.0:197.0, mean 170.2 cm, SD 9.1) and self-reported weight (min-max 42.0:180.0, mean 75.7 kg, SD 15.8).

### 2.4 Macro-level covariates

To investigate the effect of country-level characteristics, we use two variables widely employed in comparative social and health research – income inequality measured by net Gini coefficients (mean 32.9, SD 5.1) derived from the Standardised World Income Inequality Database (Solt, 2016) and the level of economic development measured by GDP PPP per capita (mean 19,098, SD 9958) derived from the World Bank (2017). Data on GDP are in constant 2011 international dollars, while using the PPP adjusted GDP indicator is necessary because it allows for robust cross-national comparison of economic development. Furthermore, using net rather than gross Gini coefficients is important because in countries with strong redistributive policies, gross and net income inequality are weakly correlated.

### 2.5 Statistical analysis

In order to compensate for about 9% of observations with missing information in at least one variable, we conduct a multiple imputation exercise via the MICE (Multiple Imputation using Chained Equations) package in Stata 15 (see Fantin et al., 2016), allowing for 10 sets of multiple imputations and combining them using Rubin's (1987) rules. In bivariate analysis, for comparison between self-reported and measured height we use a Bland-Altman plot which allows visualising differences between two measures against the averages of these measures (Bland and Altman, 1986).

In multivariate analysis, we use multilevel regression models which are frequently employed in comparative health research (Diez-Roux, 2000; Gugushvili et al., 2019; Irdam et al., 2016). These regressions are the most convenient statistical method to understand the effects of macro-level indicators as they allow for the simultaneous consideration of micro and macro-level variables (Maas and Hox, 2005). Of various forms of multilevel regression models, multilevel mixed-effects linear and logistic regressions are employed. These model specifications are chosen because the dependent variables take both a continuous form (normally distributed difference between self-reported and measured height) and a binary form (under- and over-reporting of height). For understanding the moderating effects of macro variables, we include in the mixed-effects regressions cross-level interactions between individual-level characteristics, on the one hand, and income inequality and economic development, on the other hand. Interaction terms indicate how each contextual variable affects the relationship between individual-level variables and height misreporting. In multilevel mixed-effects linear regression models, we describe explained variance on individual- and country-level using R-squared statistics proposed by Snijders and Bosker (1994).

## 3 Results

### 3.1 Bivariate analysis

**Fig. 1:**
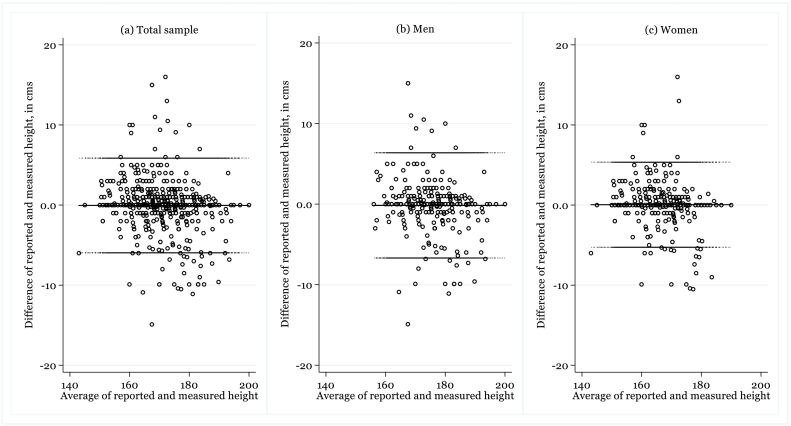
The Bland-Altman plot of differences between measured and self-reported height (in cm) against the mean of these values *Notes*: Upper and lower lines present 95% limits of agreement (LOA), where upper LOA is +1.96 SD and lower LOA is −1.96 SD from mean difference (middle line) of methods. *Source*: Authors' calculations based on data from EBRD (2016).

Horizontal lines in the Bland-Altman plot in Fig. 1 are drawn at the mean difference, and at the limits of agreement, which were defined as the mean difference plus and minus 1.96 times the standard deviation of these differences. The mean difference between reported (170.2 cm) and measured (170.3 cm) height is −0.05 cm but this difference is not statistically significant (CI95% −0.27:0.18). The Bland-Altman plots are also fitted separately for men and women, but no statistically significant differences are found between reported and measured height between genders. In Table s1 in Supplementary material, differences between these measures are also calculated separately by country. There is >20 cm difference in mean reported height between countries with the highest (Germany) and the lowest (Uzbekistan) reported height, but the estimated correlation coefficient suggests that these two measures in most countries were strongly associated (Pearson's *r* > 0.90). The direction of mean differences between reported and measured height indicates that, on average, respondents in 14 countries under-report, while in 20 countries over-report their height. In the absolute majority of these cases, however, the null hypothesis that this difference is zero cannot be rejected.

### 3.2 Multivariate analysis

In our multivariate analysis the difference scores between self-reported and measured height for each individual are used as the outcome variable and the multilevel mixed-effects linear regression models are fitted to account for both individual and contextual covariates. Results reported in Table 1 indicate that respondents' some socio-demographic and socio-economic characteristics are significantly associated with misreporting their height. First of all, in Model 2, which includes respondents' anthropometric measures, women are more likely to over-estimate their height by about 0.64 cm. Reporting higher height is also associated with individuals' age with every additional year leading to 0.02 cm of over-reporting, though this effect is only observed among women. Probably the most salient and consistent individual characteristic significantly associated with misreporting of height is respondents' residency in rural areas. In comparison to urban settlers, individuals living in rural areas are likely to overestimate their height by >1 cm among both men and women. In the pooled sample the propensity of over-reporting is also positively associated with the anthropometric measure of reported height (0.06 cm per reported 1 cm), but not with reported weight. All other individual-level variables such as education, labour market characteristics, and socio-economic position are not associated with misreporting.

**Table 1:** Multilevel analysis of individual-level predictors of height misreporting, point estimates from multilevel mixed-effects linear regression models.

	Total sample		Men		Women	
	Model 1	Model 2	Model 1	Model 2	Model 1	Model 2
Intercept	−1.75	**−10.5**	**−2.77**	−7.57	−0.89	**−8.60**
	[−3.60,0.11]	[−15.9,-5.09]	[−5.36,-0.17]	[−15.8,0.62]	[−2.51,0.72]	[−16.2,-0.95]
Gender (female = 1)	0.27	**0.64**	–––	–––	–––	–––
	[−0.13,0.68]	[0.14,1.15]	–––	–––	–––	–––
Age	**0.01**	**0.02**	0.01	0.01	**0.02**	**0.02**
	[0.00,0.03]	[0.01,0.03]	[−0.02,0.03]	[−0.01,0.04]	[0.01,0.03]	[0.01,0.04]
Settlement (rural = 1)	**1.17**	**1.13**	**1.29**	**1.36**	**1.10**	**0.99**
	[0.48,1.86]	[0.41,1.84]	[0.37,2.22]	[0.41,2.31]	[0.30,1.90]	[0.13,1.84]
Marital status (married = 1)	−0.23	−0.25	−0.27	−0.29	−0.30	−0.32
	[−0.55,0.09]	[−0.57,0.07]	[−0.71,0.17]	[−0.73,0.15]	[−0.81,0.21]	[−0.83,0.20]
Education (tertiary = 1)	0.20	0.17	0.64	0.62	−0.11	−0.12
	[−0.36,0.75]	[−0.38,0.73]	[−0.44,1.73]	[−0.45,1.70]	[−0.71,0.49]	[−0.75,0.50]
Labour market (ref. never worked)						
Unemployed	0.69	0.76	1.94	2.01	−0.03	0.05
	[−0.36,1.73]	[−0.27,1.79]	[−0.16,4.03]	[−0.09,4.12]	[−0.85,0.79]	[−0.75,0.84]
Working	0.29	0.27	1.15	1.19	−0.08	−0.12
	[−0.68,1.26]	[−0.67,1.22]	[−0.34,2.64]	[−0.24,2.62]	[−1.13,0.97]	[−1.20,0.96]
Socio-economic ladder	0.06	0.04	0.14	0.15	−0.00	−0.02
	[−0.19,0.30]	[−0.20,0.29]	[−0.15,0.44]	[−0.13,0.42]	[−0.22,0.21]	[−0.25,0.21]
Anthropometric measures						
Reported height	–––	**0.06**	–––	0.04	–––	**0.05**
	–––	[0.02,0.09]	–––	[−0.01,0.08]	–––	[−0.00,0.10]
Reported weight	–––	−0.01	–––	−0.02	–––	−0.00
	–––	[−0.03,0.00]	–––	[−0.05,0.01]	–––	[−0.02,0.01]
Snijders/Bosker R-squared: level 1	0.06	0.04	0.06	0.06	0.09	0.10
Snijders/Bosker R-squared: level 2	0.12	0.02	0.06	0.02	0.14	0.15
Number of observations/countries	750/34	750/34	344/34	344/34	406/34	406/34

*Notes*: 95% CIs are in parentheses; significant associations are shown in bold.

### 3.3 Macro-level factors and cross-level interactions

In Table 2 the standardised measures of economic development and income inequality are introduced in the pooled and split samples for men and women to test if contextual factors are associated with individuals' likelihood of misreporting their height. Based on the results in Model 1, we find no evidence that employed contextual variables are systematically and significantly associated with under or over-reporting of individuals' height. In Models 2 and 3, all individual and macro-level variables are interacted. Most of these interaction effects are not statistically significant and therefore do not appear in Table 1; however, two statistically significant effects stand out. First, in the pooled sample of men and women in countries with higher income inequality rural residents are more likely to misreport their height. Second, the effect of socio-economic position is also moderated by income inequality. Those at the higher end of social hierarchy are more likely to over-report their height in countries with higher level of net Gini coefficient.

**Table 2:** Multilevel analysis of individual and macro-level predictors of height misreporting, point estimates from multilevel mixed-effects linear regression models.

	Model 1	Model 2	Model 3
Total sample			
Macro-level variables			
Standardised GDP PPP per capita	0.33 [−0.21,0.86]	0.03 [−1.16,1.23]	0.24 [−0.26,0.75]
Standardised Gini coefficient	−0.14 [−0.67,0.40]	−0.12 [−0.69,0.45]	**−1.26** [−2.40,-0.13]
Cross-level interactions			
GDP x rural	–––	−0.43 [−1.04,0.19]	–––
GDP x socio-economic ladder	–––	0.11 [−0.06,0.29]	–––
Gini x rural	–––	–––	**1.04** [0.42,1.65]
Gini x socio-economic ladder	–––	–––	**0.18** [0.02,0.34]
Snijders/Bosker R-squared: level 1	0.06	0.06	0.09
Snijders/Bosker R-squared: level 2	0.06	0.06	0.14
Number of observations/countries	750/34	750/34	750/34

Men			
Macro-level variables			
Standardised GDP PPP per capita	0.18 [−0.45,0.81]	−0.13 [−1.47,1.20]	0.08 [−0.48,0.63]
Standardised Gini coefficient	−0.31 [−0.85,0.24]	−0.32 [−0.87,0.22]	**−1.78** [−2.77,-0.78]
Cross-level interactions			
GDP x rural	–––	0.07 [−0.68,0.81]	–––
GDP x socio-economic ladder	–––	0.06 [−0.15,0.27]	–––
Gini x rural	–––	–––	**1.41** [0.26,2.56]
Gini x socio-economic ladder	–––	–––	**0.24** [0.11,0.37]
Snijders/Bosker R-squared: level 1	0.07	0.08	0.13
Snijders/Bosker R-squared: level 2	0.07	0.08	0.22
Number of observations/countries	344/34	344/34	344/34

Women			
Macro-level variables			
Standardised GDP PPP per capita	**0.57** [0.08,1.05]	0.02 [−1.33,1.36]	0.51 [−0.01,1.02]
Standardised Gini coefficient	−0.06 [−0.60,0.48]	−0.06 [−0.64,0.53]	−0.73 [−2.15,0.68]
Cross-level interactions			
GDP x rural	–––	−0.40 [−1.28,0.47]	–––
GDP x socio-economic ladder	–––	0.17 [−0.06,0.40]	–––
Gini x rural	–––	–––	**0.92** [0.20,1.63]
Gini x socio-economic ladder	–––	–––	0.09 [−0.13,0.32]
Snijders/Bosker R-squared: level 1	0.13	0.12	0.14
Snijders/Bosker R-squared: level 2	0.20	0.17	0.21
Number of observations/countries	406/34	406/34	406/34

*Notes*: 95% CIs are in parentheses; significant associations are shown in bold; models account for all covariates shown in Table 1.

When the pooled sample is split by gender in the two lower panels of Table 2, we observe that macro-level contextual factors moderate the individual-level effects more saliently for men than for women. Although the effect of income inequality on misreporting among rural residents is also significant for women, the moderating effect of inequality for socio-economic position is only significant for men. In fact, Snijders/Bosker R-squared on level 2 is highest in Model 3 for men with interactions of income inequality and individual-level variables. Apparently, more advantaged men in countries with high income inequality are more likely to misreport their height.

**Fig. 2:**
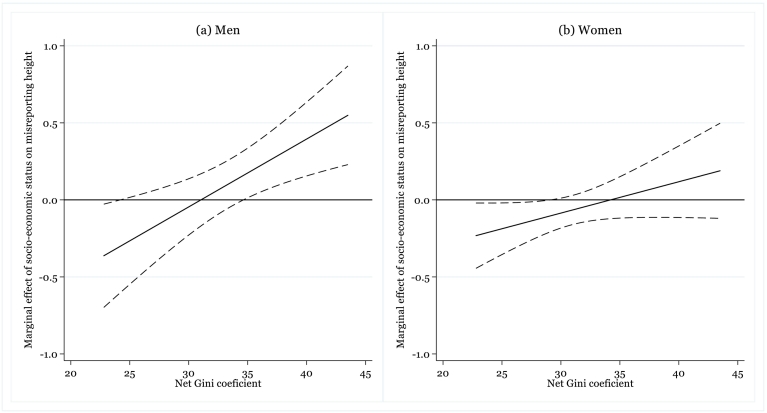
Moderating effect of income inequality on the relationship between respondents' socio-economic status and misreporting their height (in cm).

*Notes*: Marginal effects are derived from the model with the same specification as in Table 2. Dashed lines show 95% confidence intervals. *Source*: Authors' calculations based on data from EBRD (2016) and Solt (2016).

Because the direct interpretation of interaction terms in regression models is often misleading (Brambor et al., 2006), we graphically illustrate the marginal effect of socio-economic status and the corresponding confidence intervals across a substantively meaningful range of income inequality on macro-level. The central plot in Fig. 2 shows the effect of change in individuals' socio-economic position by one point (on the scale from 1 to 10) on respondents' misreporting their height. For instance, in countries with the higher level of income inequality (around 0.35 and above) such as Albania, Mongolia, and Turkey, four points increase in socio-economic status is predicted to lead to about 2.0 cm higher reported height when compared to individuals' measured height.

### 3.4 Under- and over-reporting and the analysis of “terminal” height

In Table 3, we investigate the associations between individual and macro-level variables and under-reporting and over-reporting of height. For this reason, we create two dummy variables which take value of 1 if respondents under- or over-evaluate their height and fit multilevel mixed-effects logistic regressions. The results for the pooled sample of men and women suggest that the type of settlement and respondents' age and reported height are the only consistently significant individual-level covariates of, respectively, under- and over-reporting of height. The macro-level part of the analysis also indicates that rural residents who live in countries with higher income inequality are less likely to under-report and more likely to over-report their height. Similarly, individuals in higher social positions are less likely to under- and more likely to over-report their height in countries with high income inequality.

**Table 3:** Multilevel analysis of individual and macro-level predictors of under-reporting and over-reporting height, odds ratios from multilevel mixed-effects logistic regression models.

	Under-reporting height (Yes = 1, No = 0)			Over-reporting height (Yes = 1, No = 0)		
	Model 1	Model 2	Model 3	Model 1	Model 2	Model 3
Intercept	**2.87**	**3.07**	**2.95**	**4.24**	**4.25**	**3.95**
	[1.33,6.20]	[1.32,7.14]	[1.30,6.69]	[1.61,11.20]	[1.56,11.55]	[1.56,10.02]
Individual level characteristics						
Gender (female = 1)	0.69	0.65	0.67	1.41	1.42	1.44
	[0.42,1.12]	[0.40,1.08]	[0.41,1.09]	[0.89,2.26]	[0.89,2.27]	[0.90,2.30]
Age	0.99	0.99	0.99	**1.02**	**1.02**	**1.02**
	[0.98,1.01]	[0.98,1.01]	[0.98,1.01]	[1.01,1.03]	[1.01,1.03]	[1.01,1.04]
Settlement (rural = 1)	**0.29**	**0.18**	**0.15**	0.68	0.71	1.12
	[0.12,0.66]	[0.07,0.47]	[0.05,0.42]	[0.28,1.66]	[0.27,1.84]	[0.42,3.00]
Marital status (married = 1)	1.12	1.15	1.16	0.77	0.76	0.77
	[0.75,1.67]	[0.76,1.74]	[0.77,1.74]	[0.52,1.14]	[0.51,1.13]	[0.51,1.14]
Education (tertiary = 1)	1.17	1.10	1.08	1.08	1.08	1.18
	[0.66,2.07]	[0.62,1.97]	[0.61,1.92]	[0.61,1.91]	[0.61,1.91]	[0.66,2.09]
Labour market (ref. never worked)						
Unemployed	0.80	0.76	0.80	1.18	1.18	1.17
	[0.41,1.57]	[0.38,1.51]	[0.40,1.59]	[0.63,2.22]	[0.63,2.23]	[0.62,2.20]
Working	1.07	1.05	1.04	0.66	0.65	0.66
	[0.58,2.00]	[0.56,1.99]	[0.55,1.95]	[0.36,1.20]	[0.36,1.20]	[0.36,1.22]
Socio-economic ladder	0.97	1.01	1.01	0.96	0.96	0.92
	[0.86,1.10]	[0.89,1.16]	[0.88,1.15]	[0.85,1.10]	[0.84,1.09]	[0.81,1.05]
Anthropometric measures						
Reported height	0.98	0.98	0.98	**1.04**	**1.04**	**1.04**
	[0.95,1.01]	[0.95,1.01]	[0.95,1.01]	[1.01,1.07]	[1.01,1.07]	[1.01,1.07]
Reported weight	1.01	1.00	1.01	0.99	0.99	0.99
	[0.99,1.02]	[0.99,1.02]	[0.99,1.02]	[0.97,1.00]	[0.97,1.00]	[0.97,1.00]
Macro-level variables						
Standardised GDP PPP per capita	1.16	1.32	1.26	**1.82**	1.61	**1.70**
	[0.73,1.85]	[0.52,3.37]	[0.78,2.03]	[1.08,3.07]	[0.59,4.36]	[1.02,2.86]
Standardised Gini coefficient	1.18	1.05	**2.45**	0.99	0.99	**0.38**
	[0.75,1.86]	[0.65,1.71]	[1.17,5.14]	[0.59,1.64]	[0.59,1.67]	[0.17,0.86]
Cross-level interactions						
GDP x rural	–––	**4.00**	–––	–––	0.90	–––
	–––	[1.54,10.36]	–––	–––	[0.35,2.31]	–––
GDP x socio-economic ladder	–––	**0.83**	–––	–––	1.04	–––
	–––	[0.71,0.97]	–––	–––	[0.89,1.21]	–––
Gini x rural	–––	–––	**0.22**	–––	–––	**3.19**
	–––	–––	[0.08,0.62]	–––	–––	[1.19,8.55]
Gini x socio-economic ladder	–––	–––	0.91	–––	–––	**1.17**
	–––	–––	[0.82,1.02]	–––	–––	[1.03,1.31]
Variance on country level	1.05	1.12	1.08	1.44	1.45	1.37
	[0.51,2.19]	[0.53,2.38]	[0.51, 2.31]	[0.74,2.83]	[0.72,2.89]	[0.70, 2.70]
Number of observations/countries	750/34	750/34	750/34	750/34	750/34	750/34

*Notes*: 95% CIs are in parentheses; significant associations are shown in bold.

In our main analytical sample, age of respondents ranges from 18 to 94. However, height is only constant from the age of about 25 till the age of 50 which is followed by rapid age-related shrinking (Cline et al., 1989). To minimize biological factors causing differences in the reporting of height in our sample, in regressions presented in Table 4 we limit our analytical sample to the “terminal” height cohorts who are aged between 25 and 50. With this new sample specification the number of individuals is reduced by more than half, which makes it difficult to observe any statistically significant associations. Nonetheless, the results indicate that the reported weight is negatively associated with height misreporting. In addition, as was the case in the main analysis, we find that income inequality moderates the effect of socio-economic position and rural settlement on misreporting individuals' height and these associations are only significant among men.

**Table 4:** Multilevel analysis of individual and macro-level predictors of height misreporting, individuals aged 25–50, point estimates from multilevel mixed-effects linear regression models.

	Men			Women		
	Model 1	Model 2	Model 3	Model 1	Model 2	Model 3
Intercept	−3.87	−3.57	−4.12	−5.91	−5.86	−5.82
	[−12.97,5.22]	[−12.18,5.04]	[−11.8,3.61]	[−18.9,7.05]	[−18.8,7.09]	[−18.5,6.90]
Individual level characteristics						
Age	0.02	0.02	0.04	0.01	0.02	0.02
	[−0.03,0.07]	[−0.03,0.07]	[−0.01,0.08]	[−0.02,0.05]	[−0.02,0.05]	[−0.02,0.05]
Settlement (rural = 1)	0.47	0.52	**0.78**	0.16	0.21	0.23
	[−0.16,1.10]	[−0.15,1.20]	[0.02,1.54]	[−0.72,1.05]	[−0.67,1.09]	[−0.89,1.34]
Marital status (married = 1)	0.02	−0.03	−0.04	−0.13	−0.17	−0.14
	[−0.55,0.59]	[−0.69,0.63]	[−0.62,0.54]	[−0.69,0.43]	[−0.71,0.38]	[−0.73,0.44]
Education (tertiary = 1)	0.86	0.87	1.05	−0.50	−0.37	−0.47
	[−0.51,2.23]	[−0.47,2.21]	[−0.28,2.38]	[−1.17,0.18]	[−1.16,0.42]	[−1.14,0.21]
Labour market (ref. never worked)						
Unemployed	0.27	0.21	−0.17	−0.52	−0.50	−0.51
	[−2.36,2.90]	[−2.31,2.73]	[−2.34,1.99]	[−1.43,0.38]	[−1.41,0.40]	[−1.43,0.40]
Working	0.84	0.80	0.73	0.23	0.10	0.22
	[−1.20,2.89]	[−1.18,2.78]	[−1.19,2.64]	[−0.85,1.32]	[−0.84,1.05]	[−0.80,1.25]
Socio-economic ladder	0.20	0.20	0.05	−0.12	−0.11	−0.08
	[−0.18,0.58]	[−0.18,0.59]	[−0.18,0.27]	[−0.43,0.19]	[−0.37,0.16]	[−0.32,0.16]
Anthropometric measures						
Reported height	0.01	0.01	0.02	0.04	0.04	0.04
	[−0.04,0.06]	[−0.04,0.06]	[−0.03,0.06]	[−0.05,0.13]	[−0.05,0.13]	[−0.05,0.12]
Reported weight	−0.02	−0.02	−0.02	−0.00	−0.00	−0.00
	[−0.05,0.02]	[−0.05,0.02]	[−0.05,0.01]	[−0.03,0.02]	[−0.03,0.02]	[−0.03,0.02]
Macro-level variables						
Standardised GDP PPP per capita	0.17	−0.28	0.04	**0.59**	−0.44	**0.66**
	[−0.28,0.62]	[−2.35,1.79]	[−0.39,0.48]	[0.05,1.13]	[−2.29,1.40]	[0.10,1.21]
Standardised Gini coefficient	**−0.54**	**−0.55**	**−2.62**	−0.00	−0.07	0.55
	[−0.80,-0.28]	[−0.83,-0.27]	[−4.26,-0.97]	[−0.56,0.55]	[−0.62,0.48]	[−1.18,2.29]
Cross-level interactions						
GDP x rural	–––	0.04	–––	–––	−0.21	–––
	–––	[−0.76,0.84]	–––	–––	[−1.18,0.75]	–––
GDP x socio-economic ladder	–––	0.09	–––	–––	0.24	–––
	–––	[−0.33,0.51]	–––	–––	[−0.13,0.60]	–––
Gini x rural	–––	–––	**0.97**	–––	–––	0.41
	–––	–––	[0.22,1.71]	–––	–––	[−0.54,1.36]
Gini x socio-economic ladder	–––	–––	**0.38**	–––	–––	−0.13
	–––	–––	[0.07,0.70]	–––	–––	[−0.43,0.17]
Snijders/Bosker R-squared: level 1	0.12	0.12	0.19	0.05	0.07	0.06
Snijders/Bosker R-squared: level 2	0.12	0.12	0.19	0.01	0.03	0.02
Number of observations/countries	159/34	159/34	159/34	184/34	184/34	184/34

*Notes*: 95% CIs are in parentheses; significant associations are shown in bold.

## 4 Discussion

With this study we provide new cross-national evidence of the quality of data on self-reported height in social and health surveys, which is often used to derive one of the most relevant measures of population health – BMI. Overall, our bivariate analysis suggests that on the aggregate level reported and measured height estimates are not significantly different from each other. This indicates that self-reported height is a good approximation of actual height and it could be used as a proxy anthropometric measure in health research and practice. Nonetheless, in our multivariate analysis, after adjusting for individuals' socio-demographic and socio-economic covariates, we identified that being female, older, living in rural areas, and reporting higher height were all significantly and positively associated with the propensity of misreporting one's own height. One explanation why women and rural settlers are more likely to misreport their height is that men and those who live in urban areas are getting their height measured more often. For instance, many of the countries included in the analysis have, or once had, men-only military conscription, which requires height measurement as a routine part of more general medical assessment (Schmidt et al., 1995).

Based on the results of this study, it is also possible to adjust BMI estimates of large societal groups in the considered countries. For instance, women and those who live in rural areas constitute, respectively, 53% and 37% in our analytical sample, which comes very close to the United Nations Population Division's estimates (United Nations, 2014). Both women and rural residents over-estimate their height by, respectively, 0.6 cm and 1.2 cm. This alteration would increase the estimated level of BMI for the general population by around 0.54 points, which would mean about a 2.1% increase in BMI score. Furthermore, these calculations are likely to underestimate the real levels of BMI as women are also known to under-report their weight (Gorber et al., 2007). The higher actual weight of women would make the effect of their over-reported height even more pronounced in calculations of accurate BMI scores. If we make an assumption that women and rural residents also underreport weight to the same extend as they over-report height, this would further imply increasing BMI score by 3.7%.

Some of the effects of individual-level characteristics on self-reported height are moderated by the macro-level characteristics of countries in which survey respondents reside. While we did not find evidence that net Gini coefficient and GDP PPP capita are directly associated with misreporting of height, our results suggest that individuals' reported socio-economic status, particularly among men, is positively related to over-reporting their height in more unequal societies. Although we cannot assert what are the exact mechanisms behind this association, income inequality has been linked with mental illness, violence, imprisonment, lack of trust, and drug abuse, among other detrimental societal outcomes (Wilkinson and Pickett, 2009a, Wilkinson and Pickett, 2009b). We can speculate that in countries where income inequality is high, the intensity of social comparison between better- and worse-off individuals is also stronger (Präg et al., 2014). This in turn might imply that more advantaged individuals in the higher end of social hierarchy are likely to overestimate their height. Interestingly the effect is primarily observed among men, which suggest that there are some gender-specific mechanisms behind this association. One explanation is that in post-socialist countries (making up the majority in our sample) men were more adversely affected by social, economic, and political transformations and increasing inequality (Azarova et al., 2017; Doniec et al., 2019), which could also strengthen the role of social comparison.

This study has a number of limitations which warrant cautious interpretation of the findings. First, although the pooled sample across countries is reasonably big (750 individuals), the sample sizes for individual countries are rather small, which does not allow making any generalisations regarding misreporting of height in specific countries. The goal of the study, however, was to identify the general patterns in self-reporting of height regardless of idiosyncratic country differences and the random selection of PSUs for measuring height further mitigates the problem of representation. Second, both self-reported and measured height were entered in the dataset in the same format (centimetres and millimetres), but some errors could have occurred in rounding of numbers by respondents for their self-reported height. This is partially confirmed by the distribution of height variable actually measured by survey administrators being closer to a normal distribution than that of self-reported height (see Fig. s1 in Supplementary material). Third, one of the main rationales of this study was to understand the role of self-reported height in correctly estimating BMI rates, but this task cannot be comprehensively fulfilled without analysing also the misreporting of weight. Unfortunately, LITS does not provide relevant information, but some alternative cross-national surveys which include information on individuals' actual weight can be used in future studies.

## 5 Conclusions

Based on the presented analysis and discussion, we can draw three main conclusions. First, individuals in most instances provide accurate data on their height. This is confirmed by the analysis of individuals from the randomly selected small territorial units across the large number of societies in Europe and Central Asia. Second, certain socio-demographic and socio-economic characteristics of individuals are associated with misreporting of height. Adjusting for this bias would lead to higher average BMI scores, and a higher share of individuals in populations who are overweight and obese. Third, the effect of individual-level covariates on validity of self-reported height can be moderated by country-level characteristics such as income inequality. Based on our results, future studies on anthropometric measures derived from survey data should also take into account contextual environment in which individuals live.

## Data Availability

The dataset analysed in the current study is openly available from the European Bank for Reconstruction and Development: https://www.ebrd.com/cs/Satellite?c=Content&cid=1395236498263&d=Mobile&pagename=EBRD%2FContent%2FContentLayout.

## Appendix A Supplementary data

Supplementary material Image 1
