# Supplementary material for: Inequality, validity of self-reported height, and its implications for BMI estimates: An analysis of randomly selected primary sampling units' data
Source: Prev Med Rep. 2019 Aug 16;16:100974. doi: 10.1016/j.pmedr.2019.100974 (PMC6715954; doi:10.1016/j.pmedr.2019.100974)
Supplement: Supplementary file 1 — Supplementary material [file mmc1.docx]

Table s1: Country-level characteristics of the measured and self-reported height

| *Country* | Number of observations | Reported (R) height | Measured height | Correlation coefficient | Difference between reported and measured height | P-value of t-test of difference |
| --- | --- | --- | --- | --- | --- | --- |
| Germany | 15 | 183.7 | 183.8 | 0.99 | –0.13 | 0.959 |
| Bosnia and Herzegovina | 20 | 179.8 | 180.5 | 0.91 | –0.70 | 0.767 |
| Serbia | 19 | 178.3 | 177.9 | 1.00 | 0.34 | 0.929 |
| Italy | 20 | 176.6 | 173.0 | 0.72 | 3.56 | 0.229 |
| Albania | 20 | 176.4 | 182.6 | 0.91 | –6.20 | 0.005 |
| Tajikistan | 16 | 174.6 | 176.1 | 0.78 | –1.51 | 0.636 |
| Czechia | 20 | 174.4 | 174.2 | 0.99 | 0.18 | 0.954 |
| Latvia | 19 | 173.3 | 172.0 | 0.98 | 1.32 | 0.668 |
| Montenegro | 19 | 173.0 | 172.9 | 1.00 | 0.11 | 0.960 |
| Poland | 20 | 172.3 | 172.3 | 1.00 | –0.05 | 0.977 |
| Turkey | 22 | 171.6 | 170.7 | 0.98 | 0.91 | 0.647 |
| Bulgaria | 20 | 171.5 | 171.5 | 0.99 | –0.04 | 0.990 |
| Kosovo | 20 | 171.4 | 171.2 | 0.98 | 0.19 | 0.937 |
| North Macedonia | 37 | 171.3 | 171.5 | 0.99 | –0.27 | 0.918 |
| Russia | 66 | 171.2 | 173.2 | 0.90 | –1.99 | 0.222 |
| Estonia | 20 | 171.0 | 165.2 | 0.95 | 5.80 | 0.383 |
| Greece | 13 | 170.4 | 170.2 | 0.99 | 0.23 | 0.956 |
| Cyprus | 20 | 169.9 | 166.5 | 0.87 | 3.40 | 0.167 |
| Romania | 20 | 169.7 | 169.3 | 0.87 | 0.38 | 0.903 |
| Slovenia | 20 | 169.5 | 168.9 | 0.97 | 0.57 | 0.794 |
| Georgia | 20 | 168.8 | 168.2 | 0.86 | 0.65 | 0.806 |
| Belarus | 18 | 168.8 | 167.8 | 0.99 | 0.94 | 0.668 |
| Lithuania | 40 | 168.4 | 167.8 | 0.96 | 0.55 | 0.812 |
| Croatia | 15 | 168.3 | 167.4 | 0.98 | 0.91 | 0.773 |
| Moldova | 17 | 168.1 | 167.6 | 0.98 | 0.53 | 0.855 |
| Ukraine | 16 | 168.1 | 168.1 | 1.00 | –0.06 | 0.981 |
| Hungary | 37 | 167.2 | 167.7 | 0.99 | –0.54 | 0.812 |
| Kazakhstan | 20 | 166.6 | 166.7 | 1.00 | –0.10 | 0.974 |
| Armenia | 18 | 164.8 | 166.9 | 0.95 | –2.17 | 0.524 |
| Slovak Republic | 22 | 164.7 | 164.4 | 0.99 | 0.35 | 0.881 |
| Azerbaijan | 20 | 163.7 | 163.8 | 0.94 | –0.10 | 0.929 |
| Kyrgyz Republic | 20 | 163.5 | 163.4 | 0.97 | 0.05 | 0.979 |
| Mongolia | 21 | 163.4 | 162.4 | 0.90 | 1.00 | 0.679 |
| Uzbekistan | 20 | 162.6 | 162.7 | 1.00 | –0.05 | 0.984 |
| In total | 750 | 170.2 | 170.3 | 0.95 | –0.05 | 0.925 |

*Source:* Authors’ calculations based on data from EBRD (2016)

Figure s1: Kernel density estimates for self-reported and measured height (in cm)

*Source:* Authors’ calculations based on data from EBRD (2016)
